# Prismatic Adaptation Modulates Oscillatory EEG Correlates of Motor Preparation but Not Visual Attention in Healthy Participants

**DOI:** 10.1523/JNEUROSCI.1422-17.2017

**Published:** 2018-01-31

**Authors:** Martina Bracco, Domenica Veniero, Massimiliano Oliveri, Gregor Thut

**Affiliations:** ^1^Dipartimento di Scienze Psicologiche, Pedagogiche e della Formazione, Università degli Studi di Palermo, Palermo 90128, Italy,; ^2^Dipartimento NEUROFARBA, Università degli Studi di Firenze, Firenze 50139, Italy,; ^3^NeuroTeam Life and Science, Palermo 90143, Italy, and; ^4^Centre for Cognitive Neuroimaging, Institute of Neuroscience and Psychology, University of Glasgow, Glasgow G12 8QB, United Kingdom

**Keywords:** aftereffects, attention orienting, brain oscillations, EEG, motor preparation, prismatic adaptation

## Abstract

Prismatic adaption (PA) has been proposed as a tool to induce neural plasticity and is used to help neglect rehabilitation. It leads to a recalibration of visuomotor coordination during pointing as well as to aftereffects on a number of sensorimotor and attention tasks, but whether these effects originate at a motor or attentional level remains a matter of debate. Our aim was to further characterize PA aftereffects by using an approach that allows distinguishing between effects on attentional and motor processes. We recorded EEG in healthy human participants (9 females and 7 males) while performing a new double step, anticipatory attention/motor preparation paradigm before and after adaptation to rightward-shifting prisms, with neutral lenses as a control. We then examined PA aftereffects through changes in known oscillatory EEG signatures of spatial attention orienting and motor preparation in the alpha and beta frequency bands. Our results were twofold. First, we found PA to rightward-shifting prisms to selectively affect EEG signatures of motor but not attentional processes. More specifically, PA modulated preparatory motor EEG activity over central electrodes in the right hemisphere, contralateral to the PA-induced, compensatory leftward shift in pointing movements. No effects were found on EEG signatures of spatial attention orienting over occipitoparietal sites. Second, we found the PA effect on preparatory motor EEG activity to dominate in the beta frequency band. We conclude that changes to intentional visuomotor, rather than attentional visuospatial, processes underlie the PA aftereffect of rightward-deviating prisms in healthy participants.

**SIGNIFICANCE STATEMENT** Prismatic adaptation (PA) has been proposed as a tool to induce neural plasticity in both healthy participants and patients, due to its aftereffect impacting on a number of visuospatial and visuomotor functions. However, the neural mechanisms underlying PA aftereffects are poorly understood as only little neuroimaging evidence is available. Here, we examined, for the first time, the origin of PA aftereffects studying oscillatory brain activity. Our results show a selective modulation of preparatory motor activity following PA in healthy participants but no effect on attention-related activity. This provides novel insight into the PA aftereffect in the healthy brain and may help to inform interventions in neglect patients.

## Introduction

Following a right-hemispheric lesion, patients often show visuospatial attention and motor-exploratory biases away from contralesional hemispace (Vallar, 1998; Benton and Tranel, 2003). Neglect is usually difficult to treat, but some of the lateralized deficits are alleviated by prismatic adaptation (PA) (Rossetti et al., 1998), which combines a visuomotor pointing task with prisms that displace the visual image rightward or leftward. Thus, when pointing while wearing prismatic goggles, participants initially mispoint in the direction of the prismatic shift, experiencing a visuoproprioceptive mismatch between their movement and the actual target position. Within a few trials, participants are able to adapt their movement to the new visuomotor contingencies and to compensate for the erroneous bias. As a consequence of this sensorimotor realignment, pointing movements are biased in the direction opposite to prism deviation when goggles are removed, the so-called prism aftereffect of clinical interest (Pisella et al., 2006).

Interestingly, the prism aftereffect is not merely a sensorimotor phenomenon but also extends to more complex cognitive domains (for review, see Michel, 2016). Numerous studies in healthy controls and neglect patients have reported PA aftereffects on a variety of tasks, including line bisection (Pisella et al., 2002; Schintu et al., 2014), visual search (Vangkilde and Habekost, 2010), endogenous and/or exogenous orienting of attention (Striemer and Danckert, 2007, 2010a; Nijboer et al., 2008), spatial/temporal representation (Magnani et al., 2010, 2011, 2013; Rode et al., 2010; Bultitude et al., 2013; Oliveri et al., 2013), and visually guided actions (Striemer and Danckert, 2010b).

While behavioral effects of PA have been investigated in detail, its underlying mechanisms are still debated. The most prominent account is that PA affects visuospatial attention and visuomotor functions by acting on the dorsal stream (Striemer and Danckert, 2010a). In line with this hypothesis, neuroimaging studies revealed bilateral activation of parietal and cerebellar areas during the error detection and error correction phase of prismatic adaptation regardless of prism direction (Clower et al., 1996; Luauté et al., 2006, 2009; Danckert et al., 2008; Chapman et al., 2010). The only fMRI study testing PA aftereffect reported opposite comodulation of parietal activity over the two hemispheres during a visual detection task (Crottaz-Herbette et al., 2014).

More recently, the involvement of the primary motor cortex (M1) in PA aftereffects has also been documented. Using transcranial magnetic stimulation, Magnani et al. (2014) reported increased intracortical facilitation in M1 contralateral to the prism-induced compensatory shift for both leftward- and rightward-deviating prisms. M1 involvement could be a consequence of PA-induced changes in areas connected to M1. For instance, it is conceivable that PA affects M1 via modulating parietal-M1 interactions (Schintu et al., 2016) or via its connections to the cerebellum, the latter being essential for PA as suggested by fMRI in healthy participants (Danckert et al., 2008; Crottaz-Herbette et al., 2014; Küper et al., 2014) and studies in cerebellar patients who exhibit a reduction of the prismatic aftereffect (Weiner et al., 1983; Pisella et al., 2005).

Collectively, the literature therefore indicates that PA acts on dorsal stream function, but it is unclear whether it predominantly affects attention-related or motor-related dorsal stream processes, or both. In the present study, we aimed to further probe the origin of the PA aftereffect by examining EEG changes after adaptation to rightward-deviating prisms while healthy participants performed a task involving covert attention orienting to the left or right visual field, followed by preparation of a left- or right-hand motor response in the same trial. Our analyses focused on well-known EEG signatures of lateralized anticipatory attention orienting and motor preparation, namely, asymmetric changes in occipitoparietal alpha activity (Worden et al., 2000; Thut et al., 2006; Foxe and Snyder, 2011) or rolandic mu/beta activity (Pfurtscheller and Lopes da Silva, 1999; Kilavik et al., 2013; Tan et al., 2013) to distinguish between PA aftereffects on attentional visuospatial and intentional motor processes, respectively.

## Materials and Methods

### 

#### Participants

Sixteen healthy adults (9 females, 7 males, mean age = 25.62 years, SD = 4.47 years) volunteered to participate in this experiment. All participants were right handed, had normal or corrected-to-normal vision, and reported no history of neurological or psychiatric disease. Participants were financially compensated for taking part in the study. Signed informed consent was obtained from each participant at the beginning of the experiment, which was performed at the Institute of Neuroscience and Psychology, University of Glasgow. The study was performed in accordance with the Declaration of Helsinki and was approved by the ethics committee of the College of Science and Engineering, University of Glasgow.

#### Paradigm, procedure, and apparatus

Participants performed a new double-step anticipatory attention/motor preparation paradigm involving in the same trial anticipatory attention to lateralized positions (symbolically cued orienting of visuospatial attention), followed by lateralized motor preparation (with a delayed response component). In this task, a first, attentional cue guided the focus of spatial attention, whereas a second, motor preparation cue signaled whether a right- or left-hand movement had to be prepared. The two, successive postcue intervals (of 1.5 s each) allowed us to assess the EEG correlates of anticipatory attention deployment and motor preparation toward the left versus right space respectively, namely, by analyzing changes in posterior alpha and rolandic alpha/beta oscillations, our primary EEG measures of interest. Because the motor cue was presented at validly cued/attended and invalidly cued/unattended positions, it also served as a visual target, allowing the assessment of attentional effects on both behavioral and poststimulus EEG measures (i.e., behavioral responses and visually evoked potentials to the targets).

**Figure 1.**
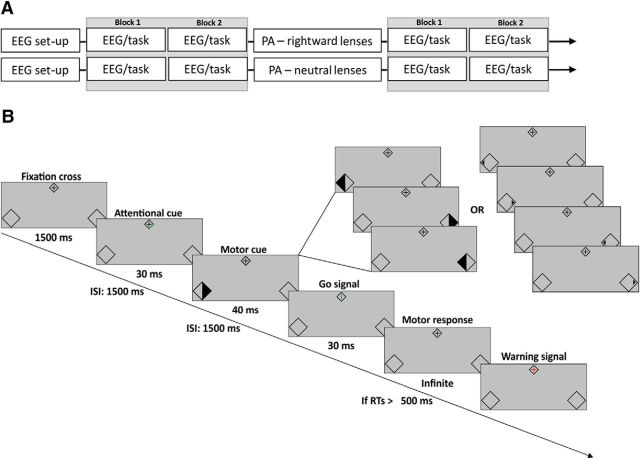
Experimental setup and paradigm. ***A***, Experimental timeline. ***B***, Experimental paradigm. Each trial started with a fixation cross, followed by an attentional cue (the bottom left or right section of the central rhombus turning green) instructing participants to covertly attend to the left or right bottom visual field placeholder. After 1500 ms, a second, motor preparation cue (big or small triangle) appeared in the left or right placeholder (80% at attended and 20% at unattended position) pointing either to the left or to the right (probability of 50%). The motor preparation cue indicated which response (left or right hand) the participants needed to prepare. After another 1500 ms, a go signal (green vertical line) instructed participants to perform the prepared action. EEG was analyzed in terms of oscillatory alpha and beta activity in the two 1500 ms Post-Cue intervals, covering anticipatory attention and preparatory motor processes to the left or right side of space, respectively, as well as in terms of visual evoked potentials to the motor cue (also serving as visual target).

All participants took part in one training session and two experimental sessions, each on a separate day. One experimental session involved PA (using prismatic lenses), whereas in the other experimental session, control (neutral) lenses were used. During the training session not involving any EEG recordings, participants were familiarized with the behavioral (attention/motor) task. This session also served for target titration. During the experimental sessions (Fig. 1*A*), participants were first prepared for EEG recordings (EEG setup). They then performed two blocks of the behavioral task lasting ∼8 min each while EEG was recorded (2× EEG; task). These two blocks served as baseline for attentional and motor preparatory EEG signatures. Afterward, participants underwent prismatic adaptation using prismatic or neutral lenses (PA; rightward or neutral lenses). After PA, EEG was again recorded while participants performed the same behavioral task for two further blocks (2× EEG; task), which served to assess PA aftereffects on the EEG signatures of interest. The order of the two experimental sessions was randomized across participants.

#### Attentional/motor task, experimental design, and analysis of behavioral data

Stimuli were presented on a CRT monitor with a 1280 × 1024 pixel resolution, a 100 Hz refresh rate, and a gray background using E-Prime (Psychology Software Tools).

Figure 1*B* illustrates the stimuli and the sequence of events per trial. Each trial began with the presentation of a central fixation cross (1.5° visual angle) inscribed into a rhombus (2° × 2°). Together with the central rhombus, two lateralized rhombi (3.5° × 3.5°) serving as placeholders were continuously displayed in the bottom left and right visual fields. After 1500 ms from trial onset, either the bottom left or the right section of the central rhombus turned green for 30 ms. This served as the attentional cue instructing the participants to covertly shift and maintain their attention toward the left or right placeholder, respectively. After 1500 ms, a left or right segment of either placeholder turned black for 40 ms (in 80% of trials at validly cued and in 20% of trials at invalidly cued position), serving both as the visual target (to assess attentional effects in both behavioral and EEG data, see below) as well as the motor preparation cue, as its form (left- or right-pointing triangle) indicated which hand the participants had to use for the upcoming motor response (for examples of motor cues, see Fig. 1*B*, top right insets). For motor cueing, the direction of the arrow pointed equally often left and rightward (50% of trials) regardless of the side of the placeholder in which the motor cue was presented. Participants were instructed to prepare a left or right index finger movement according to the motor cue direction as soon as this appeared but were asked to withhold the response for 1500 ms, until the fixation cross turned into a green vertical line for 30 ms (go signal). To encourage movement preparation before the go signal, speeded response execution was emphasized and a red cross was presented in the central rhombus as a warning signal if no response occurred within the first 500 ms after the go signal, in which case the trial was aborted and a new trial started.

The task consisted of a total of 232 trials before and 232 trials after PA, divided into 2 blocks of 116 trials each (Fig. 1*A*). In 200 of the 232 trials per preblocks/postblocks, we presented large attentional targets/motor cues that covered a full half of the placeholder (Fig. 1*B*, top right insets). In the remaining 32 trials, we used smaller attentional targets/motor cues that consisted of small left or right segments of the placeholder rhombi turning black (Fig. 1*B*, top far right insets), leading to small leftward- or rightward-pointing triangles (0.5° visual angle), and which were presented in 50% of trials at validly cued and 50% of trials at invalidly cued positions. For these small targets, luminance contrast with the background was titrated during the training session for each participant to give rise to perithreshold performance with a behavioral advantage for cued stimuli compared with uncued stimuli (mean detection accuracy valid trials = 0.75; invalid trials = 0.55). Using this design, we could control via behavioral measures inferred from the small-target/cue trials that participants shifted attention as instructed (because small stimuli were not at ceiling, i.e., led to clear attentional benefits/costs), and at the same time had enough large-target/cue trials (*n* = 100 per smallest condition cell) to analyze EEG with a good signal-to-noise ratio (small target/cue trials were excluded from EEG analysis because they are difficult to perceive and hence likely associated with uncertainty about what hand to choose for motor preparation).

Participants were seated on a comfortable chair at a distance of 57 cm from the screen. The distance was kept constant throughout the session using a chin rest. Participants were instructed to keep their eyes on the fixation cross throughout the experiment, shift their attention in response to the attentional cue without moving their eyes, and prepare, but withhold, the speeded motor response until the go signal appeared. Participants responded with their left or right index finger by a button press on a keyboard, according to the direction indicated by the motor cue.

Behavioral data were analyzed separately for “small” and “large” target stimuli. Responses to small targets were analyzed in terms of accuracy as a function of valid and invalid attentional cueing to ensure that participants engaged in the attention task. Responses to “large” targets were analyzed in terms of accuracy and reaction times for providing (descriptive) information on how well participants prepared for the motor response.

#### PA and analysis

We used a nonautomated, single-blinded PA procedure as previously described (e.g., Magnani et al., 2010, 2011, 2013, 2014; Oliveri et al., 2013). Nonautomated procedures are extensively used in the clinical setting with patients, and the procedure we used has been widely used in research, including healthy participants (Làdavas et al., 2011; Magnani et al., 2014; Calzolari et al., 2015; for other nonautomated PA procedures, see Crottaz-Herbette et al., 2014; Martín-Arévalo et al., 2016a; O'Shea et al., 2017). Participants were seated in front of a curved, horizontal Plexiglas panel (height: 30 cm, width: 72 cm, depth: 34 cm at the center and 18 cm at the periphery, distance from participant: 57 cm). The panel was placed on a tabletop between the participant and the experimenter. The concave side was facing the participant, and the convex side was facing the experimenter. The panel was transparent and graded with thin vertical lines per degrees of visual angle (120° of visual angle covered), so that the experimenter could read out the participants' pointing accuracy per trial: rightward-pointing deviations from a target were scored with positive values, leftward ones with negative values.

During PA, the experimenter placed a visual target (a pen) at the top of the surface of the transparent barrier (tipping the pen on its top edge) in one of three possible positions (randomly determined on each trial): a central position (0°), 11° to the left, and 11° to the right of center. At the start of each trial, participants were asked to keep their right hand at the level of the sternum and upon target presentation to position their finger tip on the panel at target eccentricity, at a fast but comfortable speed. The experimenter recorded spatial accuracy of pointing as distance in degrees of visual angle between the target position and the final position of the participant's finger.

**Figure 2.**
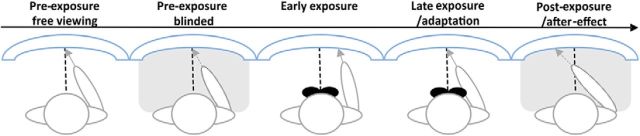
PA setup and timeline. Participants point to targets on a curved, transparent panel. Preexposure (prismatic goggles off) involves pointing in free viewing conditions (both pointing movements and targets visible) followed by occluded (blinded) pointing to visible targets. Participants were then asked to wear the googles (rightward orientation or neutral lenses) during free viewing pointing (exposure, goggles on). Adaptation is then tested immediately after exposure with blinded pointing to targets (aftereffect).

The pointing task consisted of a total of 180 trials (i.e., 60 trials for each target position) and was subdivided in three main stages: preexposure, exposure, and postexposure, with preexposure and exposure each subdivided into two further stages, leading to a total of five PA stages (Fig. 2). Preexposure consisted of 60 trials (20 trials for each pointing position). Participants performed half of the preexposure trials (i.e., 30) with visible pointing (preexposure free viewing) and half (i.e., 30) with invisible pointing (preexposure blinded). During blinded pointing, the view of the arm movement and panel was occluded by means of a cape that covered the area from neck to the edge of the panel (neither obstructing the pointing movements, nor the visibility of the top edge of the panel or the target position). During exposure, participants performed the task while wearing rightward-deviating prismatic or neutral goggles. The prisms induced a 10° shift of the visual field to the right. During exposure, participants could always see the trajectory of their movement (visible pointing) and were asked to point 90 times to targets (i.e., 30 trials per position). In the early phases of exposure (early exposure, see Fig. 2), pointing movements are typically observed to deviate to the right (with rightward-deviating goggles). In later exposure phases, this is typically compensated for by adaptation (late exposure/adaptation, see Fig. 2). In the postexposure phase, the strength of adaptation was assessed by measuring the aftereffect (usually leftward, compensatory pointing after rightward prisms) during invisible pointing (pointing movements occluded) in 30 trials (10 per target position). To limit deadaptation, participants were instructed to keep their eyes closed between PA and EEG aftereffect evaluation (postexposure invisible pointing; i.e., before starting the attention/motor task).

To probe for prismatic adaptation effects, we assessed pointing deviation from the target in visual degrees in all five stages: preexposure free viewing, preexposure blinded, early exposure, late exposure/adaptation, postexposure/aftereffect. For exposure, the first and second half of trials were analyzed separately because these are typically associated with differential effects when prismatic lenses are used (early rightward bias with rightward lenses, later compensation for this bias) (e.g., Magnani et al., 2014). To statistically test for PA effects with prismatic lenses compared with neutral lenses, we conducted a 2 × 5 repeated-measures ANOVA with Exposure type (Prism vs Neutral) and Time (5 PA phases) as within-subjects factor. Simple tests were conducted to break down main effects and interaction where appropriate.

#### EEG recording and preprocessing

EEG was continuously recorded during the task with 1000 Hz sampling rate from 62 Ag/AgCl sintered electrodes mounted on an elastic cap according to the International 10–10 system (BrainAmp, Brain Products). An additional electrode was positioned on the outer canthus of the left eye to record eye movements (when referenced to Fp1), whereas AFz and TP9 served as reference and ground, respectively. All impedances were kept <5 kΩ.

EEG data were analyzed using BrainVision Analyzer2 (BrainProducts) and FieldTrip toolbox (Oostenveld et al., 2011) (http://www.ru.nl/fcdonders/fieldtrip/) in MATLAB version 7 (The MathWorks). EEG was bandpass filtered offline from 0.5 to 80 Hz and rereferenced to the average of all channels. A band-stop filter was then used to remove 50 Hz activity. An independent component analysis was performed to remove eye blinks and muscle artifacts. EEG data were then segmented into 4000 ms epochs, starting 1000 ms before and ending 3000 ms after the first (attentional) cue (hence spanning 1500 ms into the post-motor cue period). Based on visual inspection, trials with further artifacts were rejected. Trials with small motor cues were not included in the EEG analysis. Finally, data sampling rate was reduced to 512 Hz for analysis.

The experimental design gave rise to 200 EEG trials for each of the four main conditions (Pre vs Post × Prism vs Neutral lenses), equally divided in 100 leftward-pointing and 100 rightward-pointing attentional cues, and 100 leftward-pointing and 100 rightward-pointing motor cues. From this set of trials, we discarded on average 9% of trials due to errors, slow responses, and EEG artifacts (9 ± 5.3%). Analyses were therefore based on averages of *n* = 91 trials per smallest condition cell (leftward or rightward orienting, and lefthand or righthand motor preparation).

#### EEG: time frequency analyses

For each participant, condition, and trial, time-frequency analyses were performed using fast Fourier transform for all frequencies ranging from 2 to 40 Hz, using a Hanning taper with a fixed 500 ms sliding time window moving in steps of 20 ms. The power was averaged over trials for each block of recording (Pre-/Post-Prism, Pre-/Post-Neutral). Analyses were separated to cover the epochs of anticipatory attention shifts (i.e., −200 to 1500 ms from the attentional cue onset) and of motor preparation, respectively (i.e., −200 to 1500 ms from motor cue onset). No baseline correction was applied for analysis in the frequency domain. The analyses were performed on the EEG correlates of either attention orienting or motor preparation in two steps, using the following: (1) a nonselective cluster-based analysis taking into account the whole scalp data; and (2) a planned analysis within electrodes of interest (EOIs). Both analyses were inspired by prior literature (for a recent example, see Marshall et al., 2015). Analysis 1 did not inform Analysis 2 at any stage, and hence were performed independently.

##### EEG correlates of attentional shift.

For each participant, condition, and time point, trials were averaged separately for attentional left and attentional right cues. Data were examined for EEG indices of attentional modulation by contrasting attention right and attention left trials (*Power*_Attention right_ − *Power*_Attention left_) per electrode (e.g., Marshall et al., 2015), which were then interrogated in regards to differential changes across conditions (see Statistical analyses). To normalize data, a common denominator was created to divide the data by the average over attention left and right trials of all conditions (e.g., Marshall et al., 2015), consisting here of exposure type (Prism and Neutral condition) and time (Pre- and Post-PA). To evaluate prismatic adaptation effects on attention, EEG analysis focused on activity between 8 and 12 Hz. This frequency band was predefined in line with many previous studies reporting modulation of posterior alpha activity with spatial attention deployment (for examples, see Worden et al., 2000; Thut et al., 2006; for review, see, e.g., Foxe and Snyder, 2011).

##### EEG correlates of motor preparation.

For each participant, condition, and time point, trials were averaged separately for left and right motor preparation cues. Data were then analyzed in terms of differential motor preparatory signals between left-hand and right-hand preparatory trials (*Power*_Right Hand_ − *Power*_Left Hand_) per electrode across conditions, in analogy to the analysis described above. Again, a common denominator was calculated to normalize data by dividing by the average over motor left and right trials across all conditions, that is, exposure types (Prism and Neutral) × time (Pre- and Post-PA). We analyzed activities in both the alpha/mu (8–12 Hz) and beta band (16–25 Hz), as both these frequency bands are known to be modulated by unimanual motor preparation over rolandic sensors (Pfurtscheller and Lopes da Silva, 1999; Kilavik et al., 2013; Tan et al., 2013).

##### Statistical analyses.

Statistical analyses on the above data were conducted separately for attentional and motor cue periods and frequency bands of interest (alpha and beta bands) as follows in two steps.

First, we set up cluster-based permutation statistics, including all electrodes (Maris and Oostenveld, 2007) to probe the interaction effect of interest, namely, a differential effect of intervention (Pre vs Post) depending on exposure type (Prism vs Neutral lenses) on the attention orienting and/or motor preparatory signals. The cluster-based statistics were computed over the time periods from 200 to 1000 ms for the attentional cue period, and 500–1200 ms for the motor preparatory period in the respective frequency ranges of interest (8–12 Hz, 16–25 Hz). For the cluster-based statistics, dependent-sample *t* tests were run for the contrasts of interest, that is, either on Post − Pre-Prism versus Post − Pre-Neutral (for exploring the interaction between Exposure type [Prism vs Neutral] by Time [Pre vs Post] or on Post-Prism vs Pre-Prism as well as Post-Neutral vs Pre-Neutral, for exploring the associated simple effects of Time per Exposure type when appropriate). Clusters of adjacent data points in space were defined by means of a clustering algorithm using a threshold of *p* < 0.025 (two-sided *t* test). The cluster-level test statistic was defined from the sum of *t* values of the sensors in a given cluster. Finally, clusters were evaluated in terms of statistical significance against a permutation distribution, obtained by 2500 permutations of randomly shuffling the conditions within all participants.

Second and in line with previous studies, we ran an additional analysis calculating modulation indices by attention orienting/motor preparation over posterior and central EOIs (e.g., Thut et al., 2006; Vukelić et al., 2014; Marshall et al., 2015; Wang et al., 2017), previously shown to reliably capture spatial attention deployment and motor preparation, respectively. An attentional modulation index (AMI) and a motor preparation index (MPI) were calculated per hemisphere by averaging EEG power changes over EOIs. EOIs were defined as the groups of electrodes in either the left or right hemisphere that showed the strongest average alpha/beta modulation by attention orienting/motor preparation when collapsed across all conditions (see also Marshall et al., 2015). In analogy to previous literature, these electrodes corresponded to posterior, occipitoparietal electrodes for calculation of the attention orienting index (P3/P5/P7/PO3/PO7/O1, P4/P6/P8/PO4/PO8/O2) and central electrodes for the motor preparation index (C3/CP3, C4/CP4). AMI and MPI were then calculated according to the following formula: (*Power*_Contralateral_ − *Power*_Ipsilateral_)/[common denominator] (Marshall et al., 2015), where contralateral and ipsilateral refer to the attentional focus with respect to the EOIs for the AMI, and to the hand the participants were instructed to move for the MPI. The common denominator refers to the average of contralateral versus ipsilateral changes across all conditions, that is, exposure type (Prismatic and Neutral condition) and time (Pre- and Post-PA). For both AMI and MPI, positive index values indicate a modulation of power in the direction expected from prior studies on attentional orienting and motor preparation, namely, a contralateral decrease and ipsilateral increase in power (in which case both numerator and denominator are negative). This index therefore indicates the degree of modulation observed within each hemisphere, allowing to test per hemisphere whether PA affected these modulations (the index would converge to 0 if there were no difference in power between contralateral and ipsilateral conditions). We probed whether the AMI and/or MPI are differentially affected by intervention (Pre vs Post) depending on Exposure type (Prism vs neutral) and hemisphere using a repeated-measures ANOVA with factors Exposure type (Prism vs Neutral), Time (Pre vs Post), and Hemisphere (Left vs Right).

#### Bayes factor (BF) analysis

To further inform the interpretations of our results, we calculated a BF for all statistical comparisons pointing to a null effect (*p* > 0.05) (Rouder et al., 2009). Unlike inferential statistics, which do not provide information about the null hypothesis, the Bayesian approach allows a quantification of how strong the evidence is for the alternative or the null hypothesis. To this end, we compared the magnitude of the PA-induced effects (Post-PA − Pre-PA) to changes occurring in the Neutral condition (Post-Neutral − Pre-Neutral). Our alternative hypothesis was that changes induced by PA (Post-PA − Pre-PA) are significantly different from the neutral condition, whereas the null hypothesis was that the two conditions are equivalent. Specifically, the BF was estimated setting the prior on effect size following a Cauchy distribution with a scale factor of 1 (Rouder et al., 2009). Despite the fact that evidence is continuous, BF < 1/3 can be considered as strong evidence in favor of the null hypothesis, BF > 3 as strong evidence in favor of the alternative hypothesis, whereas 1/3 < BF < 3 indicates data insensitivity (i.e., support for neither hypothesis) (Dienes, 2014).

#### Target-locked event-related potentials (ERPs)

To investigate whether PA aftereffects could manifest as a gain modulation of visual responses (poststimulus attention effect), rather than in preparatory, prestimulus activity, we analyzed ERPs locked to the visual target (also serving as motor cues) (only large targets included). For each participant and condition, EEG was low-pass filtered at 30 Hz and then segmented in 600 ms epochs, from 100 ms before to 500 ms after target presentation. All epochs were baseline corrected to 100 ms prestimulus activity and averaged over blocks of recording in each condition (Pre-/Post-Prism, Pre-/Post-Neutral). P1 and N1 peaks were then extracted as the most prominent positive and negative peaks over parieto-occipital electrodes (PO7 and PO8) within the 70–150 ms (P1) and 130–230 ms (N1) intervals after target onset, and analyzed for attentional and PA modulation, in line with previous studies (Eimer, 1994; Martín-Arévalo et al., 2016a).

##### Statistical analysis.

For each component of interest (P1 and N1), changes in peak amplitude and latency were analyzed through repeated-measures ANOVAs testing the factors Exposure type (Prism vs Neutral), Time (Pre vs Post), Cueing (Valid vs Invalid), Target position (Left vs Right), and Laterality (Contralateral vs Ipsilateral to the target position).

## Results

### PA: expected leftward bias after adaptation to rightward-shifting lenses

**Figure 3.**
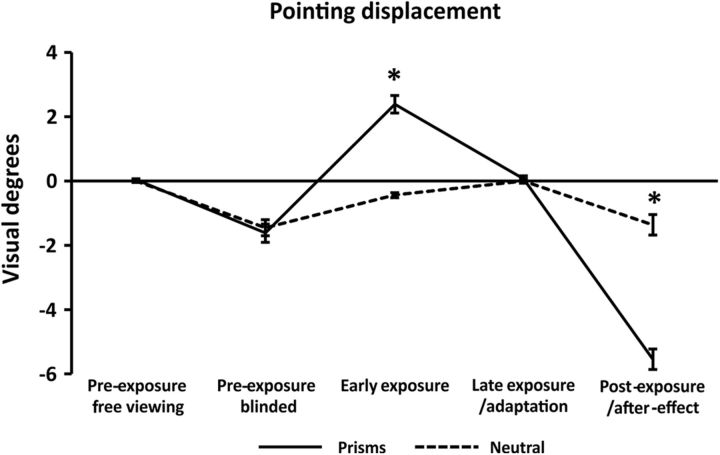
PA pointing displacement. Mean pointing displacement (expressed in degrees of visual angle) throughout the PA procedure (preexposure free viewing/preexposure blinded, early and late exposure, aftereffect) is plotted for each condition. Solid line indicates pointing when wearing real (prismatic) lenses (prismatic goggles). Dotted line indicates pointing with neutral lenses (neutral goggles). Negative values indicate a leftward-pointing displacement. Positive values indicate a rightward displacement. Error bars indicate SEM (standard error of the mean). *p < 0.001, significant difference between conditions.

Analysis of pointing displacement during PA revealed the expected pattern (Fig. 3). When wearing rightward-shifting lenses (solid line), participants showed an initial rightward-pointing deviation during early exposure (positive deflection) that was compensated for in the late exposure stage. This is explained by adaptation, given that postexposure pointing was associated with an aftereffect characterized by a leftward overshoot (negative deflection in Fig. 3). No such effects were observed with neutral lenses (dashed line). This was statistically supported by a 2 × 5 repeated-measures ANOVA revealing significant main effects of Exposure type (*F*_(1,15)_ = 5.75, *p* = 0.03, ηp^2^ = 0.28) and Time (*F*_(4,60)_ = 118.43, *p* < 0.001, ηp^2^ = 0.89) and a Exposure type × Time interaction (*F*_(4,60)_ = 104.93, *p* < 0.001, ηp^2^ = 0.87). Two repeated-measures ANOVAs performed separately for each Exposure type (Prismatic vs Neutral lenses) both showed significant main effects of Time (Prismatic, *F*_(4,60)_ = 173.45, *p* < 0.001, ηp^2^ = 0.92; Neutral, *F*_(4,60)_ = 17.01, *p* < 0.001, ηp^2^ = 0.53), each explained by different changes across PA stages. While wearing prisms, participants significantly pointed more rightward during the early exposure phase compared with the preexposure (free viewing) baseline (*F*_(1,15)_ = 74.72, *p* < 0.001, ηp^2^ = 0.83, 0.04° vs 2.38°). This bias disappeared during late exposure (*F*_(1,15)_ = 0.04, *p* = 0.83, ηp^2^ = 0.00, 2.38° vs 0.07°). In the postexposure phase, a significant leftward aftereffect was observed compared with the preexposure blinded baseline (*F*_(1,15)_ = 121.35, *p* < 0.001, ηp^2^ = 0.92, −1.62° vs −5.53°). In contrast, when wearing neutral lenses, participants showed a shift to the left in the early-exposure phase (*F*_(1,15)_ = 33.84, *p* < 0.001, ηp^2^ = 0.69, 0.0° vs −0.44°), but no significant aftereffect postexposure (*F*_(1,15)_ = 0.09, *p* = 0.76, ηp^2^ = 0.00, −1.46° vs −1.36°).

Alternatively, comparing each PA stage between the two conditions revealed no significant difference in pointing performance during preexposure (both free viewing and blinded) and late exposure (all *p* values >0.43), whereas prismatic lenses induced a rightward shift during early exposure (Prism vs Neutral: *F*_(1,15)_ = 116.77, *p* < 0.001, ηp^2^ = 0.89, 2.38° vs −0.44°) and a leftward aftereffect (Prism vs Neutral postexposure: *F*_(1,15)_ = 158.09, *p* < 0.001, ηp^2^ = 0.91, −5.53° vs −1.36°).

### Behavioral data: attentional and motor task performance

Hit rates to small targets/motor cues (indexed by correct responses to the delayed go signals) were analyzed to ensure participants did engage in attentional orienting using a repeated-measures ANOVA with the factors Exposure type (Prism vs Neutral), Time (Pre vs Post exposure), Attentional cueing (Valid vs Invalid), and Target position (Left vs Right). As expected, we found a significant main effect of Attentional cueing (*F*_(1,15)_ = 63.82, *p* < 0.001, ηp^2^ = 0.81) with more hits at validly cued than invalidly cued positions (0.83 ± 0.03 vs 0.63 ± 0.02), indicating that participants were correctly shifting their attention to the cued location. We also found significant interactions of Time × Attentional cueing (*F*_(1,15)_ = 39.31, *p* < 0.001, ηp^2^ = 0.72), Exposure type × Target position (*F*_(1,15)_ = 4.96, *p* = 0.04, ηp^2^ = 0.25), and Attentional cueing × Target position (*F*_(1,15)_ = 4.83, *p* = 0.04, ηp^2^ = 0.24). However, there was no effect in the main interactions of interest (Exposure type × Time × Attentional cueing: *p* > 0.35) and no four-way interaction with Target position (*p* > 0.35), suggesting that PA had not affected attentional processes at any target position.

Hit rates to large targets/motor cues and reaction times to go signals were analyzed to ensure that participants engaged well in motor preparation before the go signal (presented 1500 ms after the motor preparation cue). This was supported by high accuracy approaching ceiling (left motor: 0.97 ± 0.2; right motor: 0.96 ± 0.3) and fast reaction times (left motor: 291 ± 17.8 ms; right motor: 294 ± 16.7 ms). In addition, in only a small proportion of trials (4%) were participants slower than 500 ms (the response deadline). Hence, participants were engaging in the motor preparation task. Statistical analysis using repeated-measures ANOVAs on both accuracy and reaction times to large targets, taking into account Exposure type (Prism vs Neutral), Time (Pre vs Post exposure), and Hand (Left and Right) as factors, did not reveal any significant main effect or interaction (all *p* values >0.8).

### PA aftereffects on EEG signals

#### No evidence for PA to affect attention-modulated posterior alpha activity

**Figure 4.**
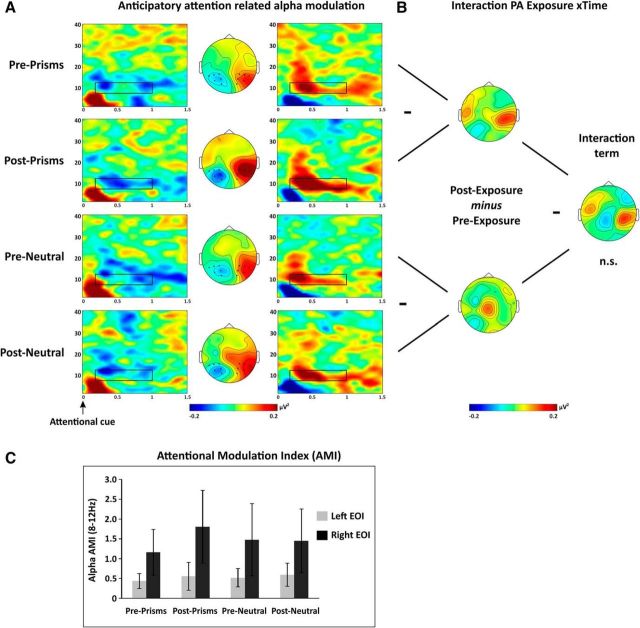
Alpha modulation by attention orienting. ***A***, Time-frequency representations of the anticipatory attention-related alpha modulation are shown separately across rows for each PA condition (Pre-/Post-Prism, Pre-/Post-Neutral) for two posterior EOIs (left and right columns) by contrasting attention right and attention left trials [(Power_Attention right_ − Power_Attention left_)/common denominator]. The electrodes included in the left and right EOIs are indicated by black dots in the central maps (P3/4, P5/6, P7/8, PO3/4, PO7/8, and O1/2). Middle column represents the topography of alpha modulation (8–12 Hz) between 0.2 and 1 s after attentional cue onset (black rectangle). ***B***, Cluster-based analysis. Difference maps of alpha modulation between conditions (8–12 Hz, 0.2–1 s Post-Cue). Raw effects are shown for each simple comparison on the left (Pre- vs Post-Prism; Pre- vs Post-Neutral) and for the Exposure × PA interaction on the right. No significant differences were identified by cluster-based statistics (all *p* values > 0.05). ***C***, EOI analysis. AMI [AMI = (Power_Attention Contra_ − Power_Attention Ipsi_)/average over all conditions] in the alpha band (8–12 Hz, 0.2–1 s) over posterior sites (P3/4, P5/6, P7/8, PO3/4, PO7/8, and O1/2). Statistical analysis revealed no significant 2 × 2 interactions. Error bars indicate SEM.

The comparison between shifts of rightward versus leftward covert attention revealed the well-known alpha signature of attention orienting. As illustrated by the time-frequency representations (Fig. 4*A*), alpha power exhibited a sustained, asymmetric modulation over left versus right occipitoparietal sites (P3/4, P5/6, P7/8, PO3/4, PO7/8, and O1/2) in accordance with the attention focus, starting 200 ms after the attentional cue and lasting up to target onset. The mirror-symmetric pattern (see map topographies in Fig. 4*A*) indicates a decrease in alpha power contralateral to the attended position and/or an increases ipsilaterally (Fig. 4*A*, topographies reflect *Power*_Attention right_ − *Power*_Attention left_ subtraction maps). Importantly, this signature was observed regardless of exposure type and time (Pre- and Post-Prism, Pre- and Post-Neutral) (compare the four rows in Fig. 4*A*).

To test for potential differences of attention-modulated alpha activity across conditions (Pre- and Post-Prism and Pre- and Post-Neutral), we first run a cluster-based permutation test (in the 8–12 Hz frequency band of interest Post-cue) taking into account all electrodes. The analysis revealed no significant cluster in the main effect of interest (Exposure type × Time interaction, see Fig. 4*B*, right middle map). Therefore, although the attention related alpha modulation seemed to be slightly accentuated Post-Prism compared with Pre-Prism (Fig. 4*B*, see top left map), this was not statistically different from pre- to post-changes in the neutral condition (Fig. 4*B*, bottom left map). To further inform this null result, we calculated the BF. This was determined separately for the left and right hemispheres considering the difference in alpha power changes (Pre vs Post) between PA and neutral condition over those occipitoparietal electrodes showing the strongest alpha power changes when collapsed across all conditions. We obtained a BF of 0.2 for the left hemisphere and a BF of 0.34 for the right hemisphere, thus providing evidence for the absence of PA effect on attentional orienting as measured by alpha power modulations.

In addition to the above cluster-based analysis approach, we ran an independent, EOI-based analysis, which further substantiated the absence of a PA aftereffect, that is, of differential effects of time (Pre vs Post), on attention-related alpha modulation as a function of Exposure type (Prism vs Neutral). We calculated an AMI (AMI = (*Power*_Contralateral_ − *Power*_Ipsilateral_)/[common denominator]) over posterior sites (P3/4, P5/6, P7/8, PO3/4, PO7/8, and O1/O2) per hemisphere and condition (see Fig. 4*C*). Positive values indicate attention modulations in the expected direction, that is, less alpha power in the contralateral versus ipsilateral condition (both numerator and denominator negative). An ANOVA testing the factors Exposure type (Prism vs Neutral), Time (Pre vs Post), and Hemisphere (Left vs Right) showed no significant main effects or interactions (all *p* values >0.12), in line with the results of the cluster-based analysis. BFs were again calculated for each hemisphere and supported a lack of PA aftereffect on attentional orienting (BF = 0.21 and 0.36 for the left and right hemisphere).

#### PA affects preparatory motor signals in the beta but not the alpha band

**Figure 5.**
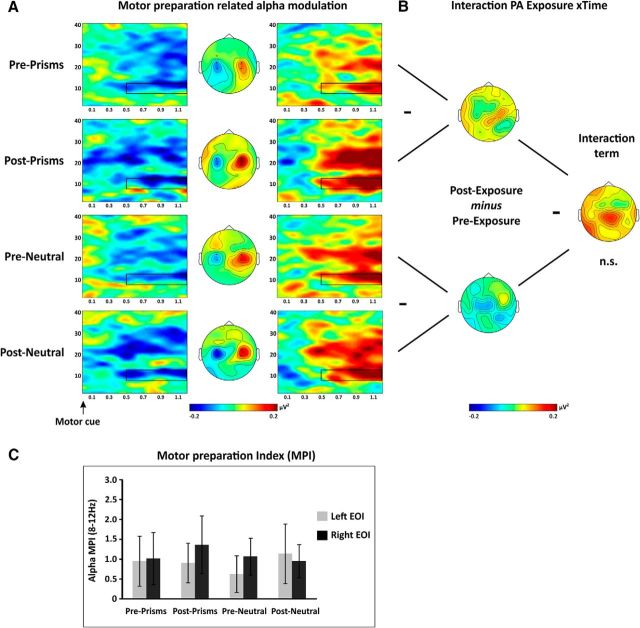
Alpha/mu modulation by motor preparation. ***A***, Time-frequency representations of the motor preparation-related alpha/mu modulation are shown separately across rows for each PA condition (Pre-/Post-Prism, Pre-/Post-Neutral) for two central EOIs (left and right columns) by contrasting right- and left-hand motor preparation trials [(*Power*_Right Hand_ − *Power*_Left Hand_)/common denominator]. The electrodes included in the left and right EOIs are indicated by black dots (C3/4, CP3/4) in the central maps. Middle column represents the topography of alpha modulation (8–12 Hz) between 0.5 and 1.2 s after motor cue onset (black rectangle). ***B***, Cluster-based analysis. Difference maps of alpha modulation between conditions (8–12 Hz). Raw effects are shown for each simple comparison on the left (Pre- vs Post-Prism; Pre- vs Post-Neutral) and for the Exposure × PA interaction on the right. No significant cluster was identified (*p* > 0.05). ***C***, EOI analysis. MPI [MPI = (Power_Hand Contra_ − Power_Hand Ipsi_)/average over all conditions] in the mu band (8–12 Hz, 0.5–1.2 s) over central sites (C3/4, CP3/4). Statistical analysis revealed no significant 2 × 2 interactions. Error bars indicate SEM.

**Figure 6.**
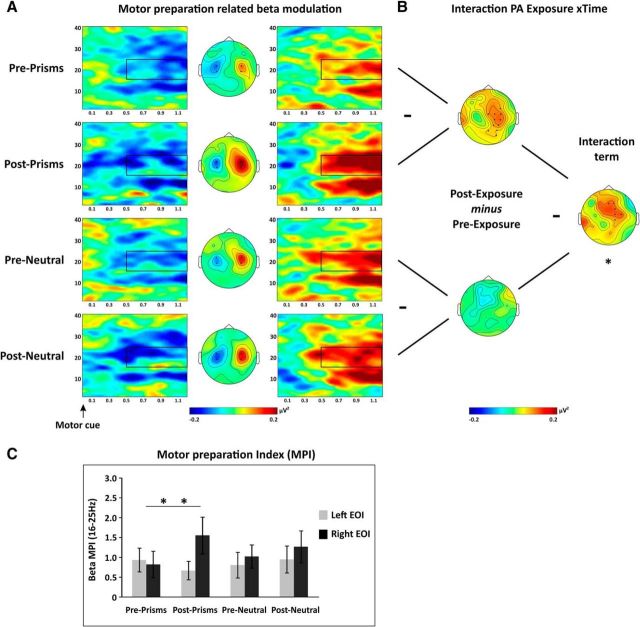
Beta modulation by motor preparation. ***A***, Time-frequency representations of the motor preparation-related beta modulation are shown separately across rows for each PA condition (Pre-/Post-Prism, Pre-/Post-Neutral) for two central EOIs (left and right columns) by contrasting right- and left-hand motor preparation trials [(*Power*_Right Hand_ − *Power*_Left Hand_)/common denominator]. The electrodes included in the left and right EOIs are indicated by black dots (C3/4, CP3/4) in the central maps. Middle column represents the topography of beta modulations (16–25 Hz) between 0.5 and 1.2 s after the cue (black rectangle). ***B***, Cluster-based analysis. Difference maps of beta modulation between conditions (16–25 Hz, 0.5–1.2 s Post-Motor cue). Raw effects are shown for each simple comparison on the left (Pre- vs Post-Prism; Pre- vs Post-Neutral) and for the Exposure × PA interaction on the right. 2 × 2 (Prism/Neutral vs Pre/Post) cluster-based permutation analyses identified a significant interaction cluster (*p* < 0.03, see black dots in right interaction map). Follow-up simple tests revealed a significant cluster (*p* = 0.008) for Pre- versus Post-Prism PA but not for Pre- versus Post-Neutral lenses (see left maps). ***C***, EOI analysis. MPI [MPI = (Power_Hand Contra_ − Power_Hand Ipsi_)/average over all conditions] in the beta band (16–25 Hz, 0.5–1.2 s) over central sites (C3/4, CP3/4). Positive values indicate the expected, contralateral versus ipsilateral modulation. Statistical analysis revealed a significant interaction of Exposure × Time × Hemisphere (*p* < 0.05). The MPI over the right hemisphere increased Post-PA (*p* = 0.015). Error bars indicate SEM. **p < 0.05.

Figures 5 and 6 show time-frequency representations of the EEG activity recorded in the motor preparatory window as difference between right-hand and left-hand movement preparation. In line with previous research (e.g., Pfurtscheller and Lopes da Silva, 1999), preparatory motor activity was associated with a distinct signature in the alpha (Fig. 5*A*) and beta bands (Fig. 6*A*). This consisted of a sustained, asymmetric modulation of alpha/beta activity over rolandic areas of the two hemispheres (i.e., most consistently observed over C3/CP3, C4/CP4) in accordance with the to-be-moved hand starting 500 ms after the motor preparation cue. The mirror symmetric pattern for both alpha and beta activity Post-Cue (Figs. 5, 6, maps) indicates that activity in these frequency bands decreased contralateral and/or increased ipsilateral to the planned movement (as topographies in Figs. 5*A*, 6*A* illustrate *Power*_Right Hand_ − *Power*_Left Hand_ subtraction maps). In analogy to the attentional epoch, these data were first analyzed by running cluster-based permutation tests, followed by EOI-based analysis to examine aftereffects of PA on motor related oscillatory signatures in the frequency bands of interest (here 8–12 and 16–25 Hz).

For the cluster-based analysis in the alpha band (Fig. 5*B*), we did not find any significant effect in the interaction of interest (i.e., Exposure type × Time; Fig. 5*B*, middle right map). Following up on this null result by calculating BF separately for the left and right hemispheres as above (but now considering the difference in alpha changes between PA and neutral condition over central electrodes showing the strongest alpha power changes across all conditions) revealed a BF of 0.2 for the left hemisphere and a BF of 1.03 for the right hemisphere, thus indicating that our data are insensitive in distinguishing null and alternative hypotheses for the right hemisphere. Additional, independent analysis of the lateralization indices of MPI (MPI = (Power_Contralateral Hand_ − Power_Ipsilateral Hand_)/[common denominator]) in the alpha band per hemisphere (i.e., over electrode pairs C3/CP3 and C4/CP4; Fig. 5*C*) also did not reveal any effects of PA on these signatures of motor preparation. The corresponding ANOVA testing the factors Exposure type (Prism vs Neutral), Time (Pre vs Post), and Hemisphere (Left vs Right) revealed no significant main effects or interactions (all *p* values >0.14; Fig. 5*C*). As for the analysis of AMI, positive values indicate that power over EOIs was modulated in expected directions (contralateral power decrease and ipsilateral power increase). Again, BF calculations pointed to a null effect over the left hemisphere (BF = 0.26) and insensitive data for the right hemisphere (BF = 0.89).

However, when considering the beta band (Fig. 6), the cluster-based permutation tests showed a significant Exposure type × Time interaction for a cluster including right central electrodes (Fig. 6*B*, middle right map; black dots illustrate the significant interaction cluster on top of the difference map) (*p* < 0.03). To break down this interaction, we ran two separate follow-up cluster-based permutation tests to compare effects of intervention (i.e., time: Pre vs Post) for Prismatic and Neutral lenses separately. The analysis revealed a significant increase of beta power after prismatic exposure over a predominantly right lateralized centroparietal cluster (*p* = 0.008) (Fig. 6*B*, top left map), whereas no clusters significantly differentiated Pre- and Post-Neutral measurements (*p* = 1) (Fig. 6*B*, bottom left map). The additional, independent analyses of MPI were in line with the cluster-based result (Fig. 6*C*). The corresponding ANOVA showed a significant Exposure type × Time × Hemisphere interaction (*F*_(1,15)_ = 4.53, *p* = 0.05, ηp^2^ = 0.23). Breaking down the interaction revealed a significant Time × Hemisphere interaction for the prism condition (*F*_(1,15)_ = 5.49, *p* = 0.03, ηp^2^ = 0.40), due to an increase in beta power modulation over the right hemisphere Post-PA relative to Pre-PA (*F*_(1,15)_ = 4.28, *p* = 0.015, ηp^2^ = 0.33), whereas no such effect emerged for the left hemisphere (*p* > 0.29). No main effects or interaction were found for the Neutral condition (*p* > 0.48; Fig. 6*C*). The increased MPI in the beta band over the right hemisphere after PA indicates enhanced motor preparatory activity in the right hemisphere, in line with the direction of the behavioral PA aftereffect (leftward compensatory shift).

### No effects of PA on attentional-modulated visual evoked potentials

Finally, visual evoked potentials to targets/motor cues were analyzed for modulation by attention and prism exposure using repeated-measures ANOVAs with the factors Exposure type (Prism vs Neutral), Time (Pre vs Post), Cueing (Valid vs Invalid), Target position (Left vs Right), and Laterality (Contralateral vs Ipsilateral hemisphere to the target position). Separate ANOVAs were conducted on peak amplitude and latency of each component of interest (P1 and N1).

#### 

##### P1.

**Figure 7.**
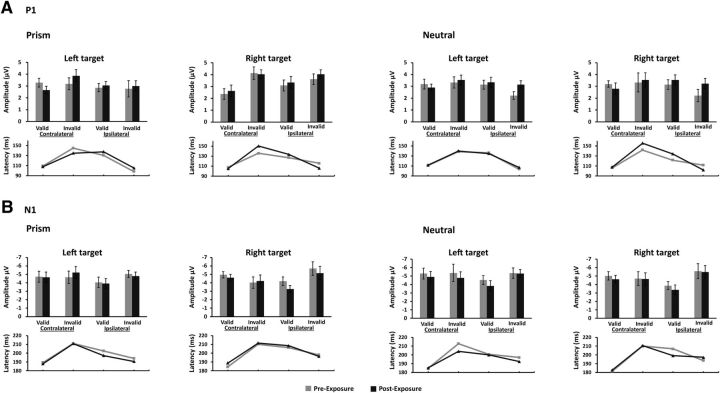
ERPs to targets/motor cues. ***A***, P1. ***B***, N1 amplitudes and latencies before and after PA (Prism condition on the left and Neutral control on the right) are shown separately for hemispheres (Ipsilateral and Contralateral to the target position), validity of attentional cueing (Valid and Invalid), and target position (Left and Right). Anticipatory attention modulated the amplitude and latency of the P1 and N1 components independently of PA. Electrodes: PO7/8.

In line with previous studies (Eimer, 1994; Martín-Arévalo et al., 2016a), the ANOVAs on P1 amplitude and latency revealed a main effect of Cueing. P1 peak amplitude was smaller in valid compared with invalid trials (*F*_(1,15)_ = 6.29, *p* = 0.02, ηp^2^ = 0.28; 3.02 vs 3.43 μV) but peaked earlier in valid than invalid trials (*F*_(1,15)_ = 5.38, *p* = 0.03, ηp^2^ = 0.30; 119.9 vs 124.3 ms). Moreover, a significant Cueing × Laterality interaction emerged for P1 latency, indicating a shorter latency over the hemisphere contralateral to the target position for the valid compared with invalid trials (Cueing × Laterality, *F*_(1,15)_ = 134.76, *p* < 0.001, ηp^2^ = 0.90; 108.2 vs 142.5 ms), and an opposite pattern for the hemisphere ipsilateral to the target position (*F*_(1,15)_ = 50.99, *p* < 0.001, ηp^2^ = 0.78; 131.70 vs 106.00 ms). No significant interactions with Exposure type × time were found either for amplitude or latency (all *p* values >0.69; Fig. 7).

##### N1.

A similar pattern of result was found for the N1 component. Its amplitude was smaller for validly cued than invalidly cued targets (main effect of Cueing: *F*_(1,15)_ = 8.10, *p* = 0.01, ηp^2^ = 0.35; −4.35 vs −4.98 μV) but peaked earlier for valid compared with invalid trials (main effect of Cueing: *F*_(1,15)_ = 14.59, *p* = 0.001, ηp^2^ = 0.49; 194.1 vs 202.5 ms). A significant Cueing × Laterality interaction pointed to smaller amplitudes for validly cued versus invalidly cued targets within the ipsilateral hemisphere (*F*_(1,15)_ = 28.33, *p* < 0.001, ηp^2^ = 0.65; −3.86 vs −5.28 μV). No other significant main effects or interaction were found either for amplitude or latency (all *p* values >0.08; Fig. 7).

## Discussion

We tested to what extent adaptation to rightward-shifting prisms can induce aftereffect on visuospatial attention orienting and/or motor preparation by examining their EEG correlates before and after prism exposure in healthy participants, compared with exposure to neutral lenses. We found significant aftereffects of PA to rightward-shifting prisms on motor preparatory activity in the beta band. Rightward PA (leading to a compensatory leftward-pointing error) enhanced preparatory rolandic beta activity over the right but not the left hemisphere (hence contralateral to the PA-induced behavioral effect). However, we did not find any PA aftereffects on visuospatial attention orienting as indexed either by attention-modulated occipitoparietal alpha activity in anticipation of a lateralized target, by attention-modulated visual evoked potentials to this target or behavioral changes. Moreover, we used two analysis approaches to test for PA aftereffects on EEG signatures of attention orienting (cluster- and EOI-based) both pointing independently to null results, and a follow-up BF analysis provided support for the null hypothesis in terms of effects on attention orienting. We therefore interpret our findings to show that rightward prisms modulate motor, but not attentional, processes.

### Differential aftereffects of PA on EEG signatures of motor preparation and visuospatial orienting

Our finding of differential PA outcomes on EEG correlates of attentional and motor processes is in line with several previous behavioral studies in healthy participants and right brain-damaged patients reporting PA effects to be related more to motor than pure attentional/perceptual functions and only detectable when the behavioral task requires an overt motor response (Farnè et al., 2002; Dijkerman et al., 2003; Ferber and Murray, 2005; Striemer and Danckert, 2010b; for review, see Striemer and Danckert, 2010a; Fortis et al., 2011; Leigh et al., 2015; Striemer et al., 2016). For example, Striemer and Danckert (2010b) found neglect patients to show a PA aftereffect only for straight-ahead pointing and manual line bisection (i.e., tasks requiring active motor responses), but not for its perceptual variant (i.e., the landmark task isolating visuospatial judgments from motor responses). Yet, it cannot be ruled out that PA affects both motor and attentional processes, and that differential aftereffects reflect different time courses of recovery (e.g., deadaptation) that could not be resolved here with our block design. In line with this view, Schintu et al. (2014) have shown that sensorimotor and visuospatial aftereffects to a single PA session last up to 35 min, but that, while the sensorimotor effects are stable, the visuospatial effects fluctuate over time. The nature of the difference between PA aftereffects on motor and attentional functions should be investigated further in future work.

A PA aftereffect at the motor level, as revealed here for the first time by means of EEG, is in accord with a growing number of transcranial magnetic stimulation studies showing PA-induced effects on motor cortex excitability (Magnani et al., 2014; Martín-Arévalo et al., 2016b; Schintu et al., 2016). This effect could represent either a direct modulation of motor cortex activity or an indirect consequence due to PA interaction with the function of connected areas. The available neuroimaging data seem to point to the latter scenario since consistently showing a sustained activation of the cerebellum and parietal cortex during PA (Luauté et al., 2006; Chapman et al., 2010). The cerebellum has an important role in movement control and preparation (Brunia, 1993), by exerting inhibitory influences on M1 via cerebello-thalamo-cortical circuits (Purzner et al., 2007). Notably, even though spectral EEG signatures of the cerebellum have not been fully elucidated, frequencies in the range of 13–25 Hz have been identified within the cerebellar cortex (Pellerin and Lamarre, 1997; Courtemanche et al., 2002; O'Connor et al., 2002); and in primates, synchronization between cerebellum and motor cortex has been observed within this frequency range (Soteropoulos and Baker, 2006). It seems therefore conceivable that the involvement of the cerebellum during PA plays an important role in inducing a change in motor cortex activity. Likewise, an influence on motor areas through the modulation of connected parietal cortex is conceivable.

Our finding of unchanged occipitoparietal EEG signatures of attentional orienting is not in support of parietal attention functions playing a pivotal role in PA aftereffect, at least for the tested population and experimental conditions (healthy participants and rightward-shifting prisms). In line with our findings, evidence for PA effects on attentional tasks in healthy participants has been so far inconclusive. Whereas some studies have reported PA effects (Berberovic et al., 2003; Striemer et al., 2006; Martín-Arévalo et al., 2016a), others failed to find behavioral effects regardless of the direction of prismatic displacement (Berberovic et al., 2004; Morris et al., 2004; Nijboer et al., 2008; Bultitude et al., 2013). On the other hand, PA to rightward-shifting prisms has repeatedly been shown to ameliorate neglect symptoms as indexed by changes in a large variety of tasks (Pisella et al., 2006; Striemer and Danckert, 2007; Nijboer et al., 2008; Rode et al., 2010; Striemer and Danckert, 2010a; Vangkilde and Habekost, 2010; Oliveri et al., 2013). To account for such generalized effects, it has been postulated that rightward-deviating prisms alleviate neglect symptoms by modulating spatial attention, possibly through a change in dorsal visual stream activity (Pisella et al., 2006; Striemer and Danckert, 2010a). Our null result in healthy participants in terms of redirection of attention to the opposite (left) space after rightward prism exposure may be linked to baseline performance in this population. Healthy participants typically show an overattention to left space at baseline (pseudoneglect) that is likely caused by right parietal dominance for spatial attention (Thiebaut de Schotten et al., 2011; Cavézian et al., 2012; Benwell et al., 2014). It is therefore conceivable that, although neglect may be alleviated by rightward prisms, causing a reorienting toward the left, neglected visual field, the use of rightward prisms may not be able to further accentuate the physiologic leftward bias in healthy participants, due to ceiling. This would be in line with a recent ERP study by Martín-Arévalo et al. (2016a) reporting leftward- but not rightward-deviating prisms to affect attention-related processes in healthy participants (i.e., attentional allocation and disengagement) using a spatial cueing task and examining ERP-changes in cue-locked N1 and target-locked P1 amplitude. In addition, it may be argued that we did not find any modulation of oscillatory signatures of anticipatory attention because PA may act at the level of exogenous, rather than endogenous, orienting of attention, as suggested by a recent fMRI study (Crottaz-Herbette et al., 2014; for a detailed model of rightward PA effects on ventral attention system, see Clarke at al., 2011). However, if so, we should have observed PA aftereffect on visual evoked potentials to targets, in particular in regard to processes indexing reorienting of attention (visual evoked potentials to targets at uncued positions), which was not the case. Overall, our data therefore do not support an attentional origin of the aftereffect of right PA in healthy participants.

### Differential aftereffects of PA on preparatory motor activity in the beta versus alpha bands

We found that rolandic beta activity was modulated by prism exposure, whereas central alpha/mu rhythms were unaffected. Despite alpha and beta activity being both considered electrophysiological markers of motor processes, they have been proposed to originate from different neural sources and subserve different functions (Salmelin and Hari, 1994; Crone et al., 1998; Pfurtscheller and Lopes da Silva, 1999; Cheyne, 2013; Tan et al., 2013). Alpha activity is observed in a wider network, including sensorimotor and parietal areas (Tzagarakis et al., 2015), and its synchronization is thought to index inhibition of task-irrelevant areas (Jensen and Mazaheri, 2010; Vukelić et al., 2014). In contrast, the rolandic beta rhythm is generated in sensorimotor areas (Ritter et al., 2009; Tzagarakis et al., 2015) and has been suggested to be more strictly related to motor functions (Baker, 2007; Veniero et al., 2011; Kilavik et al., 2013). For example, during motor imagery, rolandic alpha activity is relevant for globally inhibiting alternative motor programs (Brinkman et al., 2016), whereas rolandic beta activity is related to task-relevant movement selection (Brinkman et al., 2014, 2016). Moreover, during the cue interval of a cued, delayed motor task, the degree of rolandic beta modulation has been shown to directly reflect the extent of motor preparation (Tzagarakis et al., 2015). Therefore, in addition to further supporting a differential, functional role of rolandic alpha and beta activity, our finding of a selective modulation of beta activity suggests PA interaction with motor function at the level of movement initiation.

In conclusion, collectively, our results suggest that the aftereffects of rightward prisms in healthy participants primarily occur at the level of voluntary motor preparation but not attentional deployment, by revealing PA to selectively affect its oscillatory signatures. Our design and results could be used to further study the origin of PA aftereffects in healthy participants and neglect patients, and for informing intervention (e.g., in terms of promising target sites and protocols for adjunct neglect therapy through combining prisms with transcranial brain stimulation) (Bracco et al., 2017; O'Shea et al., 2017).
